# Development of a Qualitative Test to Detect the Presence of Organophosphate Pesticides on Fruits and Vegetables

**DOI:** 10.3390/life13020490

**Published:** 2023-02-10

**Authors:** Valentina De Luca, Luigi Mandrich, Giuseppe Manco

**Affiliations:** 1 Institute of Experimental Endocrinology and Oncology, National Research Council, Via Pietro Castellino 111, 80131 Naples, Italy; 2 Research Institute on Terrestrial Ecosystems, National Research Council, Via Pietro Castellino 111, 80131 Naples, Italy; 3 Institute of Biochemistry and Cell Biology, National Research Council, Via Pietro Castellino 111, 80131 Naples, Italy

**Keywords:** organophosphate pesticides, carboxylesterase EST2, decontamination, phosphotriesterase *Sso*Pox

## Abstract

Background: In recent decades, the use of pesticides in agriculture has increased at a fast pace, highlighting safety problems for the environment and human health, which in turn has made it necessary to develop new detection and decontamination systems for pesticides. Methods: A new qualitative test capable of detecting the presence of pesticides on fruits and vegetables by using thermostable enzymes was discovered, and the test was carried out on apples and aubergines. The contaminating pesticides were extracted from fruits with acetonitrile and analyzed with a biosensor system based on the thermostable esterase EST2 immobilized on a nitrocellulose filter. This enzyme is irreversibly inhibited mainly in the presence of organophosphates pesticides. Therefore, by observing esterase activity inhibition, we revealed the presence of residual pesticides on the fruits and vegetables. Results: By analyzing the rate of esterase activity inhibition, we predicted that residual pesticides are present on the surface of the fruits. When we cleaned the fruits by washing them in the presence of the phosphotriesterase *Sso*Pox before the detection of the esterase activity on filters, we observed a full recovery of the activity for apples and 30% for aubergines, indicating that the enzymatic decontamination of organophosphates pesticides took place. Conclusions: The reported method permitted us to assess the pesticides present on the vegetables and their decontamination.

## 1. Introduction

Pesticides are chemical substances used in agriculture for the elimination of parasitic organisms, such as animals (rodents and some birds), insects and their larvae (mites and nematodes), mollusks (slugs and snails), and plant pathogens (fungi, viruses, and bacteria), that damage cultivated plants and compromise land productivity and crop quality. Pesticides include a wide and diverse group of substances that can be classified into several categories depending on the exhibited action: the composition, the spectrum of antiparasitic activity, and specific applications. Organophosphates, carbamates, and organochlorine pesticides act directly on the central and peripheral nervous systems [1]. Pesticides act by ingestion, inhalation, or contact with molecular mechanisms that are still poorly understood [1]. Presently, with the increasing amount of pesticides used, there is concern about their adverse effects on off-target organisms, including humans, the exposure risk, and consequently the growing exposure effect [2]. Only a limited portion of pesticides that are spread in the environment achieve their intended purpose [3]. It has been estimated that in some cases, less than 0.1% of the pesticides applied to crops reach the targeted pest; the rest enter the environment gratuitously, contaminating soil, water, and air [4]. Persistence in the environment is linked to their degradability, which is determined by many factors that are unfortunately still barely known [4]. Presently, more than 600 different pesticides are commercialized, many of which are organophosphorus compounds (OPs) that are toxic for off-target organisms, including humans [5].

This group of insecticides acts on the functionality of acetylcholinesterase, an enzyme essential to nerve function in insects, humans, and many other animal species. These compounds mimic the natural substrate but irreversibly inactivate the enzyme, which is no longer capable of hydrolyzing the neurotransmitter acetylcholine, causing spasms, vasodilation, and even paralysis and death at higher doses [6]. The pesticides used indiscriminately have changed ecosystems by altering both the fauna and the flora, reducing the populations of a number of species, altering the normal behavior of animals, and depressing their reproductive capacity [7]. In fact, recent studies demonstrated that pesticide exposure is a potential risk factor for the loss of foragers, the survival of honeybees [8], the accumulation of neurotoxic compounds in fish [9], and the reproduction of earthworms [10]. The effects exerted on higher organisms by these molecules are still very complex and difficult to evaluate. A recent review identified the major risks to human health from exposure to pesticides, such as hexachlorobenzene, diethylstilbestrol, p,p’DDE, organochlorine, 2,4-dichlorophenoxyacetic acid, atrazine, and OPs, as possible damage to the immune, reproductive, endocrine, and neurological systems [11]. Moreover, the correlation between exposure to pesticides and cancer has been confirmed by an investigation conducted in California [11] in which the association appears specific for leukemia and atrazine; leukemia and 2,4-dichlorophenoxyacetic acid; leukemia and captan; and brain, testicular, and prostate cancers and atrazine. Additional studies on the toxicological properties of OPs also reported gene mutations, chromosomal aberrations, DNA damage [12], and the alteration of semen quality and sperm chromatin [13]. Moreover, the involvement of OPs in cancerogenesis and endocrine disorders has been reported [14,15]. In March 2015, 17 experts from 11 countries met at the International Agency for Research on Cancer (IARC; Lyon, France) to assess the carcinogenicity of the organophosphate pesticides tetrachlorvinphos, parathion, malathion, diazinon, and glyphosate. Insecticides tetrachlorvinphos and parathion were classified as “possibly carcinogenic to humans” (Group 2B) [16]. Consequently, the EU banned some compounds; however, there is still debate on the others (e.g., glyphosate). The increased risk for neurodegenerative diseases in adults, such as Parkinson’s disease, following the consumption of water contaminated with chlorpyrifos, diazinon, and dimethoate is particularly worrisome [17]. Recent studies have demonstrated that pesticides such as chlorpyrifos are responsible for the loss of dopaminergic neurons in young adult rats when they are exposed at the neonatal stage [18]. In addition, other studies have demonstrated that children with higher levels of trace metabolites of insecticides such as OPs are more likely to develop “Attention Deficit and Hyperactive Disorder” (ADHD), a syndrome of distracted and hyperactive children [19]. For these reasons, it has been necessary to develop an accurate and immediate method for monitoring pesticide levels. Currently, the techniques used for the OPs’ detection are gas chromatography, high-pressure liquid chromatography in combination with mass spectrometry, and recently bioassays by using fungi for environmental monitoring [20]. However, these techniques are extremely expensive and not suitable for in situ and/or real-time investigation [20,21,22,23].

Recently, an efficient alternative to common methods for detection that involves acetylcholinesterase immobilization has been proposed. The method is an automated flow-based biosensor capable of quantifying three organophosphate pesticides in milk samples: chlorpyrifos-oxon (CPO), ethyl-paraoxon (EPOx), and malaoxon (MAOx) [24]. 

The use of pesticides has enormous benefits, allowing the wider use of arable land, crop improvement, a more effective defense against infectious and parasitic diseases, and prolonged storage of agricultural and industrial products. However, these objectives have been achieved at the price of pesticide accumulation in animal tissues, plants, and water, which has deeply affected the balance of ecosystems. According to the analysis by the Environmental Working Group (EWG), since 2015, apples have been reported to be the fruit with the highest concentration of harmful substances (https://www.ewg.org/areas-focus/toxic-chemicals/pesticides, accessed on 4 July 2022). The European Food Safety Authority (EFSA) publishes an annual report on pesticide residues detected in the EU, based on information from monitoring and official checks carried out on pesticide residues in food transmitted by the 27 EU Member States and 2 EFTA countries (Iceland and Norway). The report contains data on the assessment of exposure of European consumers to pesticide residues through food. In particular, the Environmental Working Group (EWG), a non-profit advocacy agency, has released its list of the most contaminated fruit and vegetables, and apples have been ranked as the most contaminated for the fifth year in a row (Dirty Dozen™, EWG’s 2022 Shopper’s Guide to Pesticides in Produce™, at http://www.ewg.org/foodnews/dirty_dozen_guide-food-chemicals-top-12-avoid, accessed on 7 November 2022).

Because foods that have a higher presence of contaminating pesticides are fruit and vegetables, the development of biosensors that are able to find and detect the presence of pesticides on foodstuffs has ignited particular interest. A method developed recently is based on broad-specificity monoclonal antibody (MAb) for OPs against a generic hapten, *O*,*O*-diethyl *O*-(3-carboxyphenyl) phosphorothioate [25], and the method is able to detect pesticide presence on fruits and vegetables pretreated with QuEChERS [26]. 

In this work, we propose an alternative method for monitoring organophosphorus compounds based on the use of esterase 2 from *Alicyclobacillus acidocaldarius* (EST2) as a biosensor [27,28]. EST2 shows good stability in the presence of organic solvents [29,30]. It has been proven that EST2 can be quickly and easily immobilized on nitrocellulose filters and that its activity is affected by the presence of the OPs paraoxon [31], EST2 is inhibited covalently and irreversibly inhibited by OPs [31]. In a recent study, EST2 was used as a biosensor in an electrochemical system to measure the detoxifying activity of OP nerve agents by phosphotriesterases, confirming the method by HPLC and GC-FTD analysis [32]. Moreover, we used another enzyme, *SSo*Pox from *S. solfataricus* [33], a phosphotriesterase capable of hydrolyzing OPs and thus reducing their toxicity a hundredfold [33,34,35], to test its ability to degrade residual OPs on apples and aubergines. The level of detoxification was measured by using the EST2 inhibited by the residual OPs on vegetables.

## 2. Materials and Methods

Reagents. All reagents were obtained from commercial sources. 2-[4-(2-Hydroxyethyl)-1-piperazino]-ethansulfonic acid (HEPES), diethyl-p-nitrophenyl phosphate (paraoxon), acetonitrile, Fast Blue RR salt, and β-naphthyl acetate were acquired from Sigma-Aldrich, St. Louis, MO, USA. Apples and aubergines were obtained from local commercial stores; in particular, apples were of a well-known Italian brand and also of an unknown brand produced in Campania Region (Italy). The aubergines used were of an unknown brand produced in Campania Region (Italy). We confirmed that the apples and aubergines of the unknown brand are commercial products; therefore, they are subject to legal controls in order to be marketed in Italy.

Enzyme Preparation. EST2 was overexpressed in the mesophilic host *E. coli* strain BL21 (DE3) and purified as previously described by Manco et al. (1998) [36]. Purity was tested by sodium dodecyl sulfate-polyacrylamide gel electrophoresis (SDS-PAGE). Protein concentrations were estimated by the optical absorbance at 280 nm using a molar extinction coefficient of 1.34 × 10^5^ M^−1^ cm^−1^ in 40 mM sodium phosphate buffer, pH 7.1, at 25 °C, as described by Manco et al. (1998) [36]. *Sso*Pox wild type was overexpressed in the mesophilic host *E. coli* BL21(DE3) and purified as described in Merone et al. (2005) [37]. Protein concentrations were determined by using Bradford’s method (protein assay kit, Bio Rad, Hercules, CA, USA).

Pesticide extraction from the surfaces of fruits and vegetables. Pesticides were extracted from the surface of fruits and vegetables with 500 µL of 100% acetonitrile by using gentle brushing of a wet cotton swab. The acetonitrile was removed by vacuum drying (SPEEDVAC SC110, Thermo Scientific, Waltham, WA, USA), and the extracted fractions were resuspended in 120 µL methanol 30% (*v*/*v*) for one fruit. In order to set up this procedure, tests were conducted by using different amounts of solvents (acetonitrile, methanol, and ethanol). The best extraction conditions are reported above.

EST2 immobilization. The immobilized enzyme was prepared according to Febbraio et al. (2011) [31] by spotting 100 ng of freshly prepared EST2 in a 40 mM sodium phosphate buffer, pH 7.1, in delimited areas of a nitrocellulose membrane, and it dried under a heating lamp in order to optimize the immobilization process. The membranes with immobilized EST2 were stored at 4 °C up to six months, confirming high structural and catalytic stability [36].

Enzyme assay. In total, 30 µL of the extracted fraction resuspended in 30% methanol was added to the nitrocellulose filter that was previously functionalized by EST2 immobilization and incubated for 30 min at room temperature. Excess solution was removed, and the assay developed as follows: The in situ esterase activity assay of immobilized EST2 was carried out by sinking the membrane in a filtered solution of Fast Blue RR salt (0.1 g) and 2-naphthyl acetate (2 mg dissolved in 100 µL of methanol) in 1 mL solution containing 10 mM Tris/HCl buffer, pH 8.5. The esterase reaction developed 2-naphthol, which couples to Fast Blue RR salt (a diazonium salt), forming a diazo dye complex that, when insoluble, allows the detection of esterase staining activity on the membrane [38]. After 5 min, the filter was removed, washed with water, and dried. More experiments were conducted by adding to the *Sso*Pox mixture (1 µg or 2 µg) or adding sodium dodecyl sulphate (SDS) at 0.025%.

Decontamination of fruit and vegetables. Fruits and vegetables were soaked in water containing the wild-type *Sso*Pox enzyme with and without the addition of the surfactant SDS. After 60 min, the fruits were air-dried, and pesticides were extracted by the procedure described above.

## 3. Results

Apples of a well-known Italian brand and apples of an unknown brand were purchased at a local supermarket. Pesticides that might be present on the food surface were extracted with acetonitrile (100%) and used to measure EST2 residual activity, based on the color intensity of the spots obtained on the nitrocellulose membrane (as described in Methods). Furthermore, as a control, we incubated the extracted solution with 1 or 2 µg of *Sso*Pox enzyme, which is able to quickly degrade OPs [30]. 

**Figure 1:**
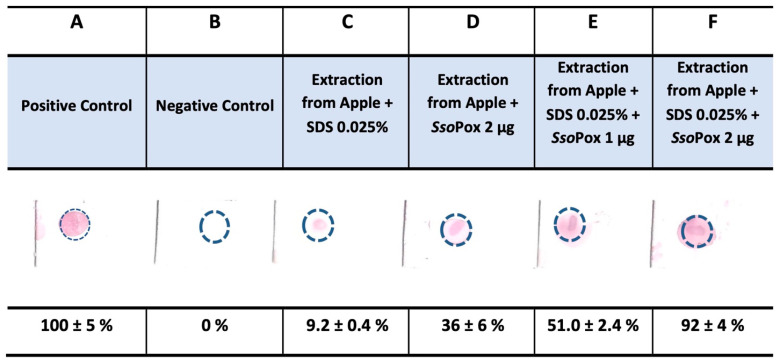
Tests on apples of a known brand. The densitometric analysis of the nitrocellulose spots obtained from apples of a known brand was made by using the GelQuantNET program (http://biochemlabsolutions.com/GelQuantNET.html). The purple color is the residual activity of EST2 enzyme. Results are the average of three different experiments.

Figure 1 depicts the analysis conducted on an apple of a known Italian brand. The result shows that the pesticides extracted from the fruits inactivated about 90% EST2 activity, measuring only 10% residual activity (Figure 1, column C). When 2 µg of *Sso*Pox (corresponding to 0.001 U_tot_) [30] was added to the mixture for 30 min, it degraded only part of the pesticides, bringing the recovery of EST2 activity on the filter to about 35% (65% inhibition; Figure 1, column D). Because it has been proven that the *Sso*Pox efficiency can be increased in the presence of the surfactant sodium dodecyl sulfate (SDS) [37], SDS (0.025% *w*/*v*) was added to the mixture under the same conditions. The results demonstrated that with 1 µg of *Sso*Pox enzyme (Figure 1 column E), the recovery of EST2 activity increased to 50%, while with 2 µg (Figure 1, column F), a 90% recovery of the activity was obtained.

**Figure 2:**
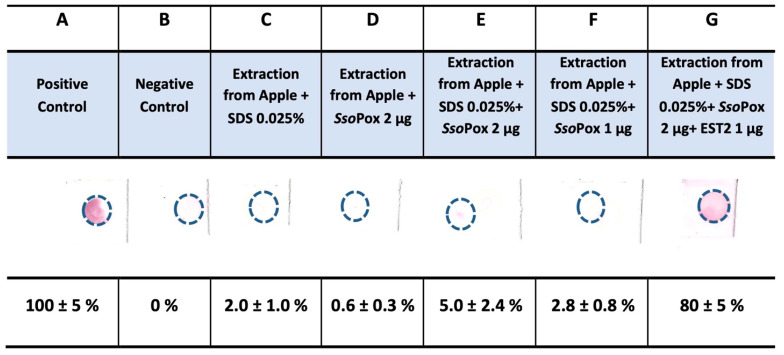
Test on apples of an unknown brand. Densitometric analysis of nitrocellulose sheet obtained by apples of an unknown origin was made by using the GelQuantNET program (http://biochemlabsolutions.com/GelQuantNET.html). The purple color is the residual activity of EST2 enzyme. Results are the average of three different experiments.

Figure 2 reports the filters obtained from apples of an unknown brand. The spots on the nitrocellulose sheet demonstrated that the pesticides extracted from the apples under the same conditions reported above completely inhibited EST2 (Figure 2, column C). Furthermore, if *Sso*Pox enzyme (1 µg or 2 µg) was added to the mixture in the presence of SDS, these putative pesticides were not degraded (5% of the recovered activity). However, when we added the decontamination mixture consisting of 1 µg of EST2 enzyme (Figure 2, column G), we obtained a recovery of the enzyme’s activity (about 80%).

The same type of analysis was performed on aubergines. According to EFSA reports, this kind of vegetable is slightly contaminated with pesticides, albeit from many different organophosphates such as dimethoate, malathion, methamidophos, omethoate, and profenofos. The choice fell on this type of vegetable because the aubergine has a smooth outer skin, similarly to apples.

**Figure 3:**
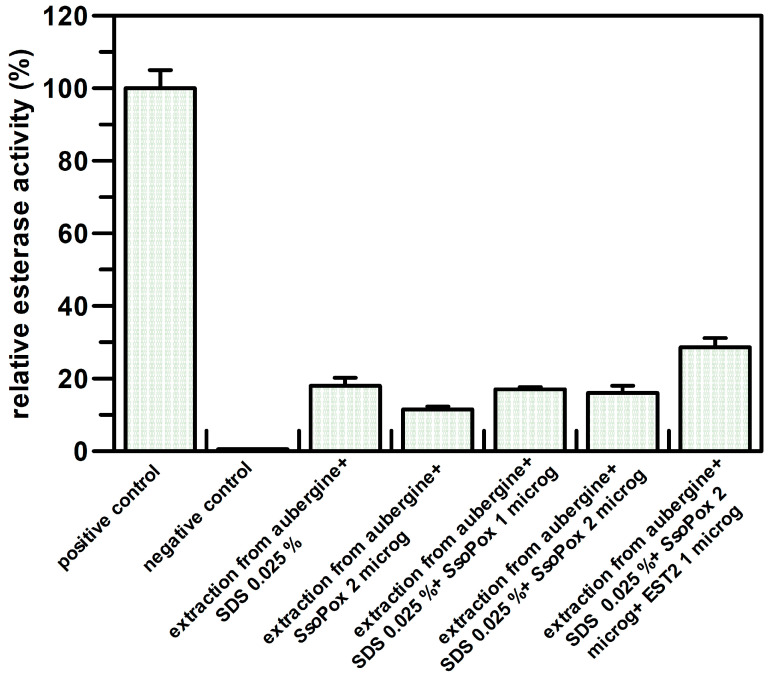
Tests on aubergines. Densitometric analysis of nitrocellulose sheet obtained by aubergines of unknown origin was made by using the GelQuantNET program (http://biochemlabsolutions.com/GelQuantNET.html). Results are reported as residual esterase activity and are the average of three different experiments.

As shown in Figure 3, in the case of the aubergine from an unknown brand, the detected EST2 inhibitors revealed were not organophosphate compounds, because by adding *Sso*Pox and SDS (Figure 3, lines 4–6; see Supplemental Figure S1) as adjuvants, the residual esterase activity detected did not change with respect to the sample treated only with SDS without the addition of *Sso*Pox (Figure 3, line 3). When we added EST2 (1 µg) to the mixture holding *Sso*Pox and SDS (Figure 3, line 7), a recovery of the esterase activity that raised to 29% was observed instead.

**Figure 4:**
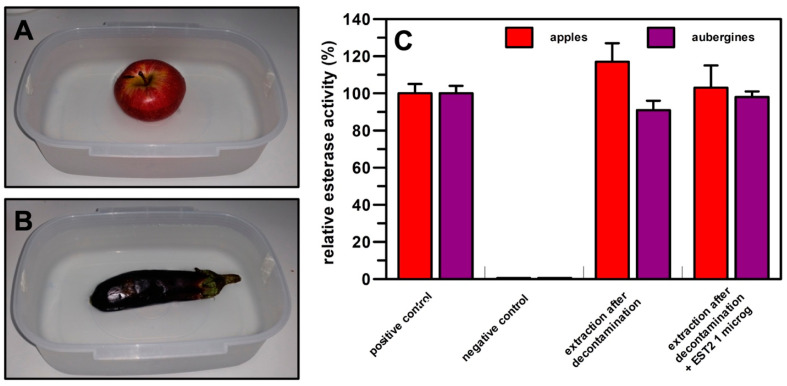
Decontamination tests on an apple (**A**) and an aubergine (**B**) treated with the *Sso*Pox enzyme for 60 min. The fruits and vegetables were washed in tap water (200 mL) containing 0.025% SDS and *Sso*Pox 1 mg/L for the time reported above; at 15 min intervals, the fruits and vegetables were extracted from the bath, air dried, and subjected to the extraction procedure of pesticides reported above. (**C**) Relative residual esterase activity determined by densitometric analysis of nitrocellulose sheet obtained by apples and aubergines after the decontamination treatment. The values are the average of three different experiments.

Based on these results, we decided to perform a wash of the fruits and vegetables in the presence of the *Sso*Pox enzyme to test, after washing, the presence of pesticides by using the previously described methodology. The fruits and vegetables were soaked in water containing *Sso*Pox and SDS (0.025%) (Figure 4A,B), and after 60 min of incubation, they were air dried, and the same extraction procedure reported above was applied. As reported in Figure 4C, pesticides present on the fruits and vegetables were removed and/or degraded after the treatment, because we observed a 100% recovery of esterase activity on the filter for apples, both in the presence or absence of EST2 (Figure 4C, lines 3, 4; see Supplemental Figure S2). For aubergines, we obtained a total recovery (approximately 98%) of esterase activity only if we pretreated the extracted mixture with the EST2 esterase (Figure 4C, line 4), whereas without pre-incubation with EST2, the esterase activity observed on the filter was about 88% (Figure 4C, line 3).

In the case of the apples of a known brand (Figure 1), by analyzing the inhibition rate of EST2 and the recovered esterase activity in the presence of *Sso*Pox, we can calculate the amount of organophosphate pesticides residing on apples. In particular, by considering an apple of about 200 gr from which it was recovered by cleaning with acetonitrile a mixture of compounds solubilized in 120 µL of 30% methanol, 30 µL of this material inhibited 90 ng of EST2, which corresponds to 2.62 nanomoles, namely the nanomoles of pesticides inhibiting EST2. From this calculation, we can deduce that 1 kg of apples contains about 52 nanomoles of pesticides. Because the average molecular weight of the most used pesticides is about 300 gr/mol, we can infer in total that about 0.016 µg of pesticides per kg of apples was present, which is lower with respect to the limit established from the EFSA (data from: https://www.efsa.europa.eu/it/topics/topic/pesticides, June 2022) of <10 µg/kg.

## 4. Discussion

To increase the production of fruits and vegetables, there has been, over time, an increase in the quantity and performance of the pesticides used, generating serious concerns both for the environment and human health. Additionally, in conventional agriculture, pesticides are extensively used against insects, fungi, and bacteria. For these reasons, national and international institutions have established limits with respect to the presence of pesticide residues in fruits and vegetables, although a way to overcome such limits is the use of different pesticides at various times during agricultural production, with the aim of keeping each of them below permitted thresholds. Even if pesticides are used at concentrations lower than those permitted, the simultaneous presence of several pesticides below the threshold value has a cumulative effect, which still generates problems for human health. One of the most dangerous effects of the absorption of pollutants involves endocrine disruptors due to their ability to interfere with the endocrine system because they mimic or interfere with the body’s hormones. These chemicals are linked with developmental, reproductive, brain, and immune problems. In addition to pesticides, there are defined endocrine disruptor chemicals such as dioxins, bisphenol A (BPA), perchlorate, perfluoroalkyl and polyfluoroalkyl substances (PFAS), phthalates, and others [39].

The possibility of detecting toxic compounds in foods is of relevant interest, and recently, several enzymatic and non-enzymatic systems have been proposed. One of these is an electro-chemiluminescent biosensor for the detection of OPs. This system is extremely sensitive, but it can be used only in a laboratory setting with a specific working station [40]. Another complex non-enzymatic electro-chemical method for detecting three OPs was based on silver nanoparticles and carbon nanotubes [41]. Among enzymatic methods, one was based on acetylcholinesterase immobilized on silica nanoparticles to detect OPs by an electro-chemical method [42]. In comparison to the others, our system is very cheap, because the required enzymes can be produced at industrial levels and are easily purified [33,36,43]. Physiological studies in the shake flasks of thermostable PLLs demonstrated that the use of galactose as an inducer increased enzyme concentrations by 4.5-fold, compared to the production obtained by induction with IPTG. By optimizing high cell density fed-batch strategies, the production and the productivity of both enzymes were further enhanced (26 folds), and the resultant fermentation processes were scalable from 2.5 L to 22.0 L. After being produced and extracted from the cells, the enzymes were first purified by a thermo-precipitation step, and the conditions were optimized by using response surface methodology. A following ultra-filtration process on 100 and 5 KDa cut-off membranes resulted in final pureness and a total recovery of both enzymes of 70.0 ± 2.0%, which is suitable for industrial applications. A proof-of-concept of this achievement was shown in the paper published by Porzio et al. [32] in which the enzymes were used in nebulized forms (in grams) [32].

Moreover, the enzymes used in this research have a long shelf life, are stable relative to many organic solvents [29,30], and can be arranged in a tool that can be easily used on the site.

In addition to our previous efforts [25,31,32,33,35], we have demonstrated a qualitative system for detecting the presence and type of pesticides on fruits and vegetables and removed them based on two enzymes: esterase EST2, which is inhibited by OPs, and phosphotriesterase *Sso*Pox, which hydrolyses OPs. We demonstrated that on the surface of fruits and vegetables, there is a “record” of the production and quality control phases of the considered vegetables. In fact, we detected and confirmed the presence of OPs in apples and aubergines (Figure 1 and Figure 3). In the case of apples of a known brand, about 90% of EST2 inactivation was observed, and after *Sso*Pox treatment, about 90% of EST2 activity was recovered (Figure 1, line F). This observation potentially indicates that these apples were prevalently treated with OPs, but a fraction of all pesticides present on the surface were not Ops because they were not hydrolysed by *Sso*Pox. In addition, they were able to inhibit EST2 (Figure 1, line F). 

In the case of apples of an unknown brand, the detected pesticides inhibit EST2, but they were not Ops, and even in the presence of *Sso*Pox, there was no recovery of EST2 activity (Figure 2, line F). When free EST2 was added to the sample, this amount of enzyme neutralized part of the pesticides, and on the filter, a residual 80% EST2 activity was observed (Figure 1, line F).

A similar effect was observed for the aubergines (Figure 3). In this case, the amount of pesticides inhibiting EST2 was higher, and only a 30% recovery of EST2 activity on the filter was observed (Figure 3).

When the apples and aubergines were detoxified by washing them in a water solution of SDS (0.025% *w*/*v*) and *Sso*Pox (1 mg/L), there were no traces of pesticides inhibiting EST2 detected on their surfaces (Figure 4). Moreover, the coupled use of the two enzymes, *Sso*Pox and EST2, allowed us to measure the amount of residual OPs pesticides present on the surface of the apples.

We are aware that one limitation of this new method is the restriction of specific OPs; however, this can be an incentive for discovering enzymes with different sensitivity to other pesticides in order to have a more complete monitoring and/or detoxification tool. From this point of view, different protein engineering approaches could allow a change in the specificity of EST2 to obtain increased sensitivity and specificity not only for detecting the OPs but also for identifying the OPs that we are dealing with. 

## 5. Conclusions

The use of pesticides in the agricultural field is necessary to respond to the growing demand for food products, but the pesticides used present serious hazards to the environment and human health. For that reason, the detection of pesticides and their biotransformation products in food is of utmost importance. It is of general interest to ensure that the amount of pesticides used in agriculture is not dangerous to human health. The simple method reported in this research is able to provide a quick response and a fast solution to controlling the presence of toxic chemicals, specifically organophosphate pesticides in vegetables.

This research represents a starting point for developing an enzyme-based biosensor system with applications in the field of food traceability as well as environmental monitoring.

## Data Availability

Not applicable.

## Supplementary Materials

The following supporting information can be downloaded at https://www.mdpi.com/article/10.3390/life13020490/s1, Figure S1: Tests on aubergine; Figure S2: Tests of decontamination on apples and aubergines.
