# Supplementary material for: Development of a Qualitative Test to Detect the Presence of Organophosphate Pesticides on Fruits and Vegetables
Source: Life (Basel). 2023 Feb 10;13(2):490. doi: 10.3390/life13020490 (PMC9958579; doi:10.3390/life13020490)
Supplement: Supplementary file 1 [file life-13-00490-s001.zip › life-2155318-supplementary.pdf]

| A | B | C | D | E | F | G |
|---|---|---|---|---|---|---|
| **Positive Control** | **Negative Control** | **Extraction from aubergines + SDS 0.025%** | **Extraction from aubergines + *Sso*Pox 2 µg** | **Extraction from aubergines + SDS 0.025% + *Sso*Pox 1 µg** | **Extraction from aubergines + SDS 0.025% + *Sso*Pox 2 µg** | **Extraction from aubergines + SDS 0.025% + *Sso*Pox 2 µg + EST2 1 µg** |
| 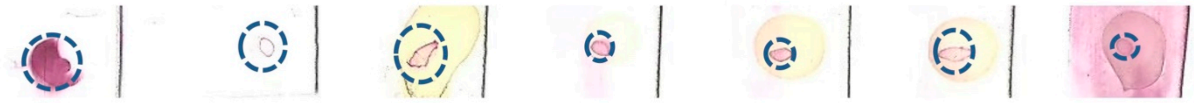 | | | | | | |
| 100 ± 5 % | 0 % | 18.0 ± 2.2% | 11.5 ± 0.4 % | 17 ± 0.1 % | 16 ± 2 % | 28.6 ± 2.5 % |

**Figure S1**. Tests on aubergines. The densitometric analysis of nitrocellulose sheet obtained from aubergines of unknown origin was made by using the GelQuantNET program (http://biochemlabsolutions.com/GelQuantNET.html). The purple color is the residual activity of EST2 enzyme. Results are the mean of three different experiments.

| | A | B | C | D |
|---|---|---|---|---|
| | **Positive Control** | **Negative Control** | **Extraction after decontamination** | **Extraction after decontamination + EST2 1µg** |
| **Apples** | 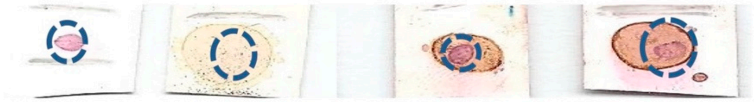 | | | |
| **Aubergines** | 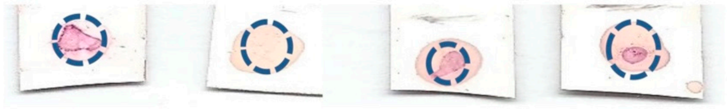 | | | |

**Figure S2**. Tests of decontamination on apples and aubergines. Fruits and vegetables were washed in tap water containing 0.025% SDS and *Sso*Pox 1 mg/L for 60 min. The densitometric analysis of nitrocellulose sheet obtained from apples and aubergines was made by using the GelQuantNET program (http://biochemlabsolutions.com/GelQuantNET.html). The purple color is the residual activity of EST2 enzyme. Results are the mean of three different experiments.
